# Improving dental disease diagnosis using a cross attention based hybrid model of DeiT and CoAtNet

**DOI:** 10.1038/s41598-025-32514-9

**Published:** 2026-01-06

**Authors:** Naira Elazab, Nermeen Nader, Yasmin Alsakar, Waleed Mohamed, Mohammed Elmogy

**Affiliations:** 1 Information Technology Department, Faculty of Computers and Information, Mansoura University, Mansoura, 35516 Dakahlia Egypt; 2 Computer Science Department, Faculty of Computers and Information, Mansoura University, Mansoura, 35516 Dakahlia Egypt

**Keywords:** Dental diagnosis, Dental X-ray scans, Data-efficient image transformer (DeiT), Convolutional attention network (CoAtNet), Cross-attention fusion, Biomedical engineering, Electrical and electronic engineering, Mathematics and computing

## Abstract

Accurate dental diagnosis is essential for effective treatment planning and improving patient outcomes, particularly in identifying various dental diseases, such as cavities, fillings, implants, and impacted teeth. This study proposes a new hybrid model that integrates the strengths of the data-efficient image transformer (DeiT) and convolutional attention network (CoAtNet) to enhance diagnostic accuracy. Our approach’s first step involves preprocessing dental radiographic images to improve their quality and enhance feature extraction. The model employs a cross-attention fusion mechanism that aligns and merges feature representations from DeiT and CoAtNet, leveraging their unique capabilities to capture relevant patterns in the data. A stacking classifier, comprising base classifiers such as support vector machines (SVM), eXtreme gradient boosting (XGBoost), and multilayer perceptron (MLP), optimizes classification performance by combining predictions from multiple models. The proposed model demonstrates superior performance, achieving an accuracy of 96%, a precision of 96.5%, 96.1% for sensitivity, 96.4% for specificity, and 96.3% for Dice similarity coefficient, thus showcasing its effectiveness in the automatic diagnosis of dental diseases.

**Supplementary Information:**

The online version contains supplementary material available at 10.1038/s41598-025-32514-9.

## Introduction

Dental X-ray image analysis and processing play a key role in diagnosing, treating, and studying the nature of dental disorders, as well as predicting dental diseases at their early stages^1^. Dental X-ray radiography is a routine tool for radiologists and a valuable resource for identifying dental disorders and issues that are difficult to detect by visual inspection alone^2^. Furthermore, manually examining an extensive collection of X-ray pictures can be time-consuming because of the low sensitivity of visual inspection and tooth structure analysis. As a result, human screening may not detect many caries. In most circumstances, an automatic computerized instrument that can aid the research process would be extremely useful. Dental image evaluation involves enhancing, segmenting, extracting features, and identifying regions to detect cavities, fractures, cysts, tumors, root canal length, and tooth growth in youngsters^3^.

Today, deep learning (DL) and machine learning (ML) approaches are widely used in digital X-ray imaging (DXRI) analysis. Convolutional neural networks (CNNs), a DL framework, are commonly used to process big datasets. Pre-trained networks like AlexNet, VGG, GoogLeNet, and Inception V3 have performed well in various experiments. CNN networks typically evolve from shallow layer networks to more complex, problem-specific networks^4^.

Accurate diagnosis of dental conditions such as cavities, fillings, implants, and impacted teeth is crucial for effective treatment and patient care. Dental radiography, a primary tool for identifying these conditions, presents unique challenges due to the high similarity between specific dental abnormalities and the fine-grained details necessary to distinguish them^5^. Traditional diagnostic methods can be time-intensive and are often subject to variability, impacting the quality of diagnosis and treatment outcomes^6^. Recent advancements in DL have opened new possibilities for enhancing diagnostic accuracy in medical imaging, including dental radiography. However, single-method models, such as standalone CNNs or transformers, may not fully capture the complex and multi-scale features needed for precise dental condition classification. This limitation highlights the need for more sophisticated models to integrate local and contextual information to improve diagnostic consistency and reliability^7^.

To address these challenges, hybrid DL models have emerged, combining the feature extraction capabilities of CNNs with the contextual understanding of transformer models. CNNs are particularly effective for identifying localized patterns, crucial in capturing the details needed to distinguish various dental conditions. However, CNNs alone may lack the broader context required to differentiate between similar-appearing abnormalities, which can lead to misclassifications.

Transformers, in contrast, excel at capturing long-range dependencies and provide a broader understanding of image context, showing significant promise in various medical image analysis tasks. Despite this potential, the integration of CNNs and transformers in a single framework for dental diagnostics remains relatively unexplored. Additionally, ensemble learning methods, which leverage the strengths of multiple classifiers, are known to enhance classification accuracy and robustness yet are rarely utilized alongside hybrid architectures in dental imaging.

This study proposes a new hybrid framework that combines the convolutional attention network (CoAtNet) and data-efficient image transformer (DeiT) architectures, leveraging a cross-attention fusion mechanism and a stacking ensemble classifier (support vector machines (SVM), eXtreme gradient boosting (XGBoost), multilayer perceptron (MLP)) to optimize feature integration and classification. The CoAtNet model uniquely combines convolutional and transformer layers, making it particularly well-suited to capture multi-scale features within dental images. By incorporating a cross-attention fusion mechanism, the framework is able to selectively align and integrate significant features from both architectures, emphasizing the most relevant information. The stacking ensemble, which includes SVM, XGBoost, and MLP classifiers, further refines the model’s decision-making, resulting in robust classification performance even for complex cases. This combination of techniques allows the model to effectively address the challenges of dental condition classification.

In this work, we present a new hybrid framework for dental condition classification designed to overcome the limitations of traditional diagnostic methods and single-model DL approaches. Our model demonstrates significant advancements in accuracy and robustness by leveraging a unique combination of CNN and transformer architectures, along with a cross-attention fusion mechanism and ensemble learning. The following key contributions highlight the innovations and improvements introduced by our approach:

- Development of a new hybrid framework combining the CoAtNet and Diet transformer architectures, specifically for dental condition classification.
- A Cross-attention fusion mechanism has been introduced to align and integrate multi-scale features from convolutional and transformer-based models, thereby improving model focus and selection.
- Implementation of a stacking ensemble classifier (SVM, XGBoost, and MLP) improves classification accuracy by combining the strengths of multiple classifiers in difficult dental diagnostic tasks.

The structure of this paper is as follows. Section 2 reviews prior work on dental disease analysis through medical imaging, focusing on two main categories of feature extraction methods: handcrafted techniques and DL-based approaches. Section 3 provides an in-depth description of the proposed framework, which is composed of five key stages: preprocessing, feature extraction, feature fusion, and classification. Section 4 details the experimental analysis, including a description of the datasets, evaluation metrics, and a discussion of the obtained results. Lastly, Section 5 concludes the study and outlines potential directions for future research.

## Related work

Disease detection and identification of dental images is a critical area in healthcare. It focuses on the early diagnosis of multi-oral conditions using advanced imaging techniques, such as panoramic X-rays, periapical images, and cone-beam computed tomography (CBCT), to identify dental diseases like tooth decay, periodontal disease, cysts, abscesses, and oral cancers. The subsequent subsections will explore the prevailing methods for identifying distinctive and significant features from dental images. These approaches can be categorized into methods based on handcrafted features (HF) and those leveraging DL-derived features.

Singh et al.^8^ presented a method for detecting and classifying dental caries based on texture features. It applied local binary pattern (LBP), gray level co-occurrence matrix (GLCM), gray level run length matrix (GLRLM), and local binary gray level co-occurrence Matrix (LBGLCM). Then, the principal component analysis (PCA) was applied to select features. AdaBoost classifier was applied to classify dental images. It achieved 99.7 % accuracy for the LBGLCM method. Yaduvanshi et al.^9^ presented a method for detecting and identifying dental oral cancer diseases based on texture features. It applied a modified local binary pattern (MLBP) to extract essential texture features. After that, SVM was applied in the classification step. This method was worked on the Mendeley dataset ^10^, which consists of 528 oral squamous cell carcinoma (OSCC) images and 696 normal epithelium oral cavity histopathological images of 400x resolution. It achieved 94.44 % accuracy.

Prajapati et al.^11^ presented a method for dental disease classification based on DL models. A labeled dataset consisting of 251 radio visiography (RVG) x-ray images of 3 different classes was used for classification. It used a very deep convolutional network 16 (VGG16) network for dental caries classification and achieved 88.46 % accuracy. Pakbaznejad et al.^12^ proposed a dental caries detection and classification method based on Convolution Neural Network (CNN). The early identification of caries is very important. Neural networks may be valuable for evaluating radiographic bone loss and generating image-based periodontal diagnostics. It achieved 85% accuracy.

Ren et al.^13^ proposed a dental caries detection and classification method. Dental caries has three types: shallow, moderate, and deep caries. The proposed method depended on a feature patch-based attention model to enhance the performance accuracy of dental caries in CBCT images. The overlapping patches from the 3D feature maps were extracted, and each patch was assigned a weight. This was important to identify the caries region. It worked on CBCT images: 167 for moderate caries and 157 for deep caries. It achieved 92% accuracy for caries classification. Vinayahalingam et al.^14^ presented a method for classifying dental caries from panoramic radiographs depending on DL techniques. This method depended on the MobileNet V2 network. 400 cropped panoramic images were used in this paper. The trained MobileNetV2 was applied to a 100-cropped PR(s) test set. It achieved 87% accuracy.

Almalki et al. ^15^ proposed a method for detecting and classifying teeth diseases. Previous studies suffered from many problems, such as experiential operation complexity, low efficiency, and user intervention at a higher level. This paper worked on four diseases: root canals, cavities, broken-down root canals, and dental crowns. It depended on the YOLOv3 DL model to identify dental abnormalities in dental panoramic X-ray images (OPG). It achieved 99.33 % accuracy. Kim et al. ^16^ proposed a method for teeth disease identification based on the DL model. It worked on five diseases: proximal caries, coronal caries or defects, periapical radiolucency, residual root, and cervical caries or abrasion. The fast region-based convolutional network (Fast R-CNN), residual neural network (ResNet), and inception models were used to learn the data. The Fast R-CNN achieved 90 % accuracy.

Using panoramic X-rays, Kong et al.^17^ constructed an ensemble model with 75% accuracy using the EfficientNet and Res2Next algorithms. Zhang et al.^18^ used a pre-trained ResNet-50 CNN to predict dental implant failure (success, failure with/without bone loss) based on 1080 X-rays from 248 patients. They achieved an area under the curve (AUC) equal to 94% for the combined model. Hasnain et al.^19^ introduced a method for diagnosing and classifying dental diseases from X-ray images. There were 126 photos in the dataset, each labeled as normal or affected. Data augmentation was first used to expand the size of the dataset. The CNN model comprises convolutional, max-pooling, flatten, dense, and output layers.

Park et al.^20^ looked into the possibility of automatically classifying dental implant sizes using two artificial intelligence techniques based on periapical radiographs. DL was used in the first method with a pre-trained VGG16 model that was optimized to extract features related to implant size from image data. Using k-means++ clustering, the second method concentrated on a feature vector obtained from important implant landmarks. Hasnain et al.^21^ assessed the effectiveness of various EfficientNet models (B0 through B7) for detecting dental diseases in panoramic radiographs. The dataset used in their study consisted of X-ray images categorized into three groups: cavities, fillings, and implants. To mitigate the issue of class imbalance, they applied the borderline synthetic minority over-sampling technique. Among the models tested, EfficientNet-B5 achieved the highest performance, surpassing other variants.

Rashid et al.^22^ proposed a DL model based on the InceptionResNetV2 architecture for the classification of seven oral diseases: gingivostomatitis, canker sores, cold sores, oral lichen planus, oral thrush, mouth cancer, and oral cancer. To support their research, they introduced a novel dataset called “Mouth and Oral Diseases,” which included images from these categories. Minoo and Ghasemi^23^ investigated the use of DL models to classify prevalent teeth conditions such as calculus, tooth discoloration, and caries. Their study leveraged three pre-trained CNN architectures—VGG16, VGG19, and ResNet50—and applied 5-fold cross-validation on a labeled dataset of dental images to ensure reliable performance. ResNet50 emerged as the best-performing model, achieving an accuracy of 95.2%, outperforming the other architectures. A summary of recent methods for dental disease diagnosis is presented in Table 1.

**Table 1:** The comparison of some previous studies.

Paper	Method type	Features Extraction	Classifier	Dataset	Disease	Accuracy (%)	Limitations
Singh et al.^8^	HF	LBP, GLCM, GLRLM, and LBGLCM	Adaboost	Private	Caries	99.7	No processing used
Yaduvanshi et al.^9^	HF	MLBP	SVM	Mendeley data	Oral Cancer	94.4	Low quality images
Prajapati et al.^11^	DL	VGG16	Softmax	RVG	Caries	88.4	Small dataset
Pakbaznejad et al.^12^	DL	CNN	Softmax	Private	Caries	85	Low accuracy
Ren et al.^13^	DL	Attention model	Softmax	Private	Caries	92	Low accuracy
Vinayahalingam et al.^14^	DL	MobileNetV2	Softmax	Private	Caries	87	Focus only on third molars
Almalki et al.^15^	DL	YOLOv3 model	Softmax	Private	Root canals, cavities, broken-down root canals, and dental crowns.	99.3	Old YOLO version
kim et al.^16^	DL	Fast RCNN	Softmax	Private	Proximal caries, coronal caries, periapical radiolucency, residual root, and cervical caries.	90	Large deviation in classes number for each tooth disease.
Hasnain et al.^21^	DL	EfficientNet Variants (B0-B7)	EfficientNet-B5	Panoramic radiographs	Cavities, Fillings, Implants	98.3	Slow inference time
Rashid et al.^22^	DL	Inception- ResNetV2	Softmax	MOD dataset	Canker Sores, Cold Sores, Oral Lichen Planus, Oral Thrush, Mouth Cancer, Oral Cancer, Gangivostomatitis	99.5	High computational cost
Minoo and Ghasemi^23^	DL	VGG16, VGG19, ResNet50	Softmax	Labeled teeth images	Calculus, Tooth Discoloration, Caries	95.2	Limited model interpretability

Despite advances in DL for dental imaging, state-of-the-art (SOTA) studies frequently have limitations that affect diagnostic accuracy and generalisability. Many existing models are based solely on CNNs or transformer architectures, which limits their ability to capture fine-grained and contextual information required for dental diagnostics. CNNs are good at extracting localized features but struggle in complex, high-similarity cases requiring more context. Transformer models, on the other hand, are excellent at modeling global dependencies but can be computationally expensive and may overlook smaller, detail-specific features. Furthermore, ensemble learning techniques are rarely used with hybrid architectures, which limits model robustness and adaptability in real-world clinical settings where diagnostic variability is a significant challenge.

Our model overcomes these limitations by combining the strengths of both CNNs and transformers via a cross-attention fusion mechanism. This hybrid approach allows the model to capture local details and more significant contextual relationships, which are critical for distinguishing between similar dental conditions such as cavities and fillings. The Cross-Attention Fusion mechanism improves feature integration by selectively aligning relevant features, ensuring that both data types are used effectively. Furthermore, the model’s robustness and classification accuracy are improved using a stacking ensemble of SVM, XGBoost, and MLP classifiers. Our model outperforms traditional SOTA methods thanks to its comprehensive approach, making it more accurate, adaptable, and clinically viable for automated dental diagnostics.

In recent years, several deep learning frameworks have been proposed for medical image classification that emphasize interpretability and multi-level feature learning. For instance, studies such as^24–26^ proposed explainable AI (XAI)-integrated pipelines using multi-stage or ensemble deep models with PCA, ELM, or multi-scale CNNs to improve both performance and clinical transparency. While these works focus on gastrointestinal and pulmonary diseases, their methodological insights—particularly the integration of feature selection and explainability—can inspire applications in dental diagnostics as well.

In the dental imaging domain, recent state-of-the-art CNN-based approaches have leveraged multi-scale and parallel CNN architectures to capture diverse lesion characteristics and improve diagnostic accuracy. Table 2 compares our proposed model to recent dental imaging studies employing hybrid or attention-based architectures. Unlike prior works that utilize fusion at bounding-box, decoder, or spatial attention levels, our model uniquely applies cross-attention fusion at the feature representation level, integrating DeiT and CoAtNet. Moreover, we deploy a stacking ensemble of diverse classifiers (SVM, XGBoost, MLP) to improve robustness—a strategy not employed in previous studies. This novel combination leads to enhanced diagnostic accuracy in dental radiographs.

**Table 2:** The comparison of attention-based and hybrid dental imaging models in recent literature.

Study	Backbone(s)	Fusion / attention strategy	Classifier / objective	Diagnostic task
Gao et al.^27^	Grouped bottleneck transformer (CNN+transformer)	Architect-level fusion	Transformer-based classifier	Tooth type classification (CBCT)
Ghafoor et al.^28^	Swin transformer + M-Net	Teeth attention block (TAB)	Segmentation head for multiclass labels	Panoramic X-ray tooth segmentation
Rezaie et al.^29^	ResNet50 + SimAM spatial attention	Spatial attention module	Softmax classifier	Radiographic disease classification
Küçük et al.^30^	YOLO + RT-DETR	Bounding-box fusion via WBF	Ensemble detection head	Impacted tooth detection (panoramic X-ray)
The proposed	DeiT + CoAtNet	Cross-attention at feature level	Stacking ensemble (SVM, XGBoost, MLP)	Dental radiograph disease diagnosis

## The proposed framework

This section describes the proposed DL framework for diagnosing dental diseases in detail. The proposed model for dental disease diagnosis employs a systematic and comprehensive methodology encompassing several critical stages: preprocessing, feature extraction, feature fusion, and classification. Initially, preprocessing is performed to enhance the quality of dental radiographic images, ensuring that relevant features are highlighted while reducing noise and artifacts. This step is crucial for optimizing the subsequent stages of analysis. Next, feature extraction is conducted using advanced techniques from the DeiT and CoAtNet, which capture intricate patterns and essential details within the images.

**Figure 1:**
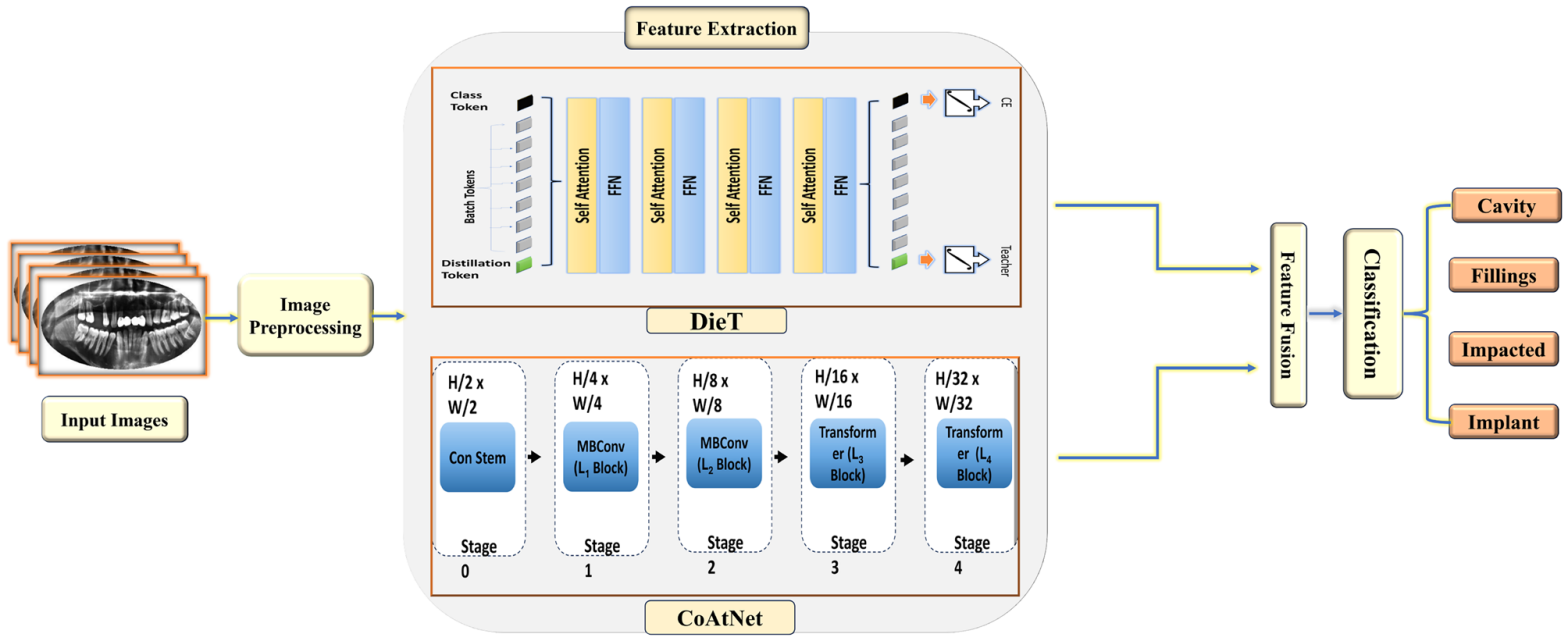
The proposed hybrid architecture showing the input image flow through patch embedding, DeiT and CoAtNet feature extraction, cross-attention fusion, and final multi-class classification using a stacking ensemble (SVM, XGBoost, MLP).

Following this, feature fusion integrates the distinctive features obtained from both models, facilitating a more robust representation of the data. Finally, a stacking classifier combines the predictions from multiple base classifiers, including SVM, XGBoost, and MLP, to ensure accurate and reliable classification of various dental conditions, such as cavities, fillings, implants, and impacted teeth. This multi-faceted approach not only leverages the strengths of each model but also enhances diagnostic performance, ultimately contributing to improved patient care. The architecture of our proposed dental disease diagnosis method is illustrated in Figure 1.

### Image preprocessing

Image preprocessing involves computational techniques that enhance digital image quality, making images suitable for feature extraction in diagnostic analysis. This framework employs several key preprocessing steps, including image normalization, adaptive histogram equalization, and optional data augmentation for improved model input quality. Each technique is detailed in the following sections.

#### Contrast limited adaptive histogram equalization (CLAHE)

CLAHE provides a more refined approach to enhance image contrast, particularly for medical imaging, where CLAHE’s localized adjustment and noise-limiting capabilities make it well-suited. It mitigates the risk of noise amplification in regions with homogeneous intensity, making it a superior choice for dental radiographs where subtle anatomical details are essential ^31^. CLAHE improves contrast in specific areas of an image while limiting the amplification of noise and artifacts. This is achieved by dividing the image into non-overlapping regions called tiles, enhancing contrast within each tile, and then seamlessly blending these tiles for a uniform appearance ^32^. Here’s how CLAHE operates:

- **Tile-Based Histogram Equalization:** The image is divided into $m \times n$ tiles (or windows), typically small non-overlapping regions. Each tile is processed independently to equalize contrast based on its unique intensity distribution. For a given tile $\omega$, the histogram $h_\omega$(i) is computed for all intensity levels i within the tile.
- **contrast Limiting:** To prevent excessive contrast in any tile, CLAHE applies a threshold known as the clip limit. Any intensity count in $h_\omega$(i) that exceeds this clip limit is redistributed across other intensity levels, limiting high-contrast enhancements that could introduce noise. Mathematically, if the pixel count $h_\omega$(i) for an intensity level i exceeds the clip limit $Clip_{lim}$, the excess is clipped:
  $$h'_w(i) = {\left\{ \begin{array}{ll} h_w(i) & \text {if } h_w(i) \le \text {Clip}_{\text {lim}} \\ \text {Clip}_{\text {lim}} & \text {if } h_w(i) > \text {Clip}_{\text {lim}} \end{array}\right. }$$
  where $\text {Clip}_{H}$ is the clipping limit set to control the maximum height of the histogram. The excess counts are then redistributed uniformly across all intensities, keeping the overall intensity distribution balanced.
- **Cumulative Distribution Function (CDF):** The modified histogram $h'_w(i)$ is used to compute the cumulative distribution function (CDF) for each tile. This CDF, $\text {CDF}_w(i)$, transforms the pixel intensities, mapping them to an enhanced range:
  $$\text {CDF}_w(i) = \sum _{j=0}^{i} h'_w(j)$$
  Using this CDF, each pixel intensity $p$ in the tile is mapped to a new intensity $p'$:
  $$p' = \frac{\text {CDF}_w(p) - \text {CDF}_{\min }}{N - 1} \times (M - 1)$$
  where $\text {CDF}_{\min }$ is the minimum value of $\text {CDF}_w(i)$ in the tile, $N$ represents the number of pixels, and $M$ is the number of intensity levels (typically 256 for grayscale).
- **Interpolation:** CLAHE seamlessly merges the tiles to create a final enhanced image. Linear interpolation between adjacent tiles ensures a smooth transition across the entire image, avoiding visible boundaries.

**Fig. 2:**
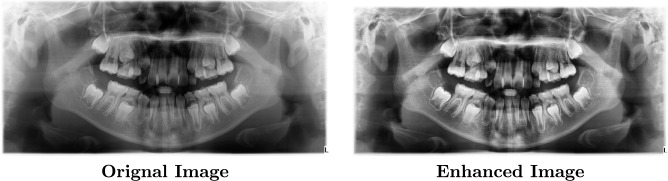
The CLAHE technique for dental image enhancement.

This approach effectively adjusts local contrast, ensuring enhanced visibility of critical features in dental radiographs without oversaturating any areas. CLAHE is particularly advantageous in medical imaging, as it emphasizes structures while reducing noise sensitivity, making it ideal for diagnostic tasks in this framework. Figure 2 indicates an example of images after applying this technique.

#### Image standardization

Image standardization helps ensure that the input images are consistent and appropriately scaled for neural network processing. This step reduces computational complexity by normalizing intensity values and standardizing the input distribution for models. Each pixel intensity I(x,y) undergoes normalization as follows:

$$I_{norm}=\frac{I(x,y)-I_{mean}}{I_{sdv}}$$

where $(I_{norm}$ is the normalized image while $I_{mean}$ and $I_{sdv}$ are the image mean and standard deviation, respectively.

#### Resizing

Standardizing image dimensions to $224 \times 224$ pixels ensures compatibility with pre-trained models such as MobileNetV2 and Swin transformer. During resizing, bilinear interpolation preserves essential spatial details, maintaining structural integrity within dental images, which is crucial for diagnostic tasks. Data augmentation techniques, such as random rotations, flips, or slight scaling, can be introduced here to increase dataset variability and improve model robustness.

- **Consistent Dimensions:** All images are resized to a standard $224 \times 224$ resolution, aligning with pre-trained model input requirements and simplifying batch processing.
- **Bilinear Interpolation:** This method preserves spatial detail, minimizing the loss of information critical for diagnosis.
- **Augmentation:** Applying augmentation techniques, such as slight rotations or brightness adjustments, increases dataset diversity and reduces overfitting risks.

Together, these preprocessing techniques ensure that dental radiographs are optimized for neural network input and enhance the robustness and accuracy of the model, contributing to a more reliable dental disease detection system.

### Feature extraction

In our proposed model, the feature extraction stage is designed to leverage the strengths of both DeiT and CoAtNet. This stage aims to generate rich and complementary feature representations from the input images, preparing them for fusion in the subsequent steps.

#### Data-efficient image transformer (DeiT)

DeiT is a specialized variant of the vision transformer (ViT) designed to be more data-efficient, utilizing a novel teacher-student distillation process. The DeiT model processes images by converting them into a sequence of image patches that can be represented as tokens. Each token is embedded and processed through self-attention mechanisms to capture spatial dependencies and contextual information ^33^.

**Fig. 3:**
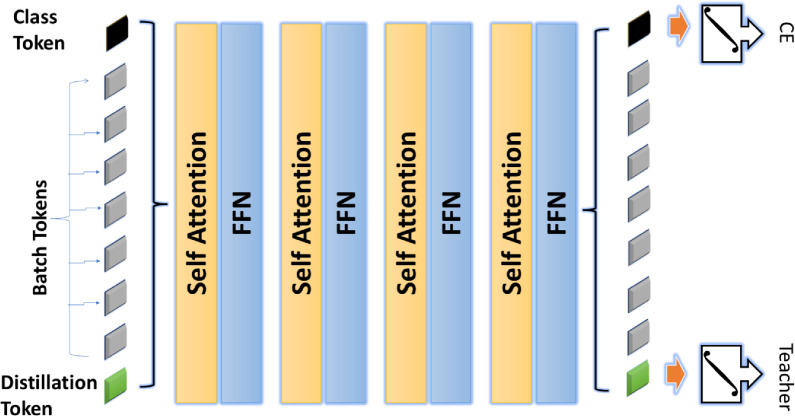
The DieT architecture.

Let the input image be denoted as $\textbf{x} \in \mathbb {R}^{H \times W \times C}$, where $H$, $W$, and $C$ represent the height, width, and number of channels, respectively. The process of tokenization in DeiT involves reshaping and embedding patches of size $p \times p$ as follows:

- **Patch Embedding:** The image is split into a sequence of $N$ patches, where
  $$N = \frac{H \times W}{p^2}.$$
- **Linear Embedding:** Each patch is linearly embedded into a vector of dimension $d$, resulting in:
  $$\textbf{z}_0 = [\textbf{x}_{cls}; E\textbf{x}_1; E\textbf{x}_2; \ldots ; E\textbf{x}_N] + E_{pos}$$
  where $\textbf{x}_{cls}$ is a class token, $E$ is the embedding matrix, and $E_{pos}$ represents the position embeddings.
- **Self-Attention Mechanism:** The self-attention layer is defined as:
  $$\text {Attention}(\textbf{Q}, \textbf{K}, \textbf{V}) = \text {Softmax}\left( \frac{\textbf{Q}\textbf{K}^{\top }}{\sqrt{d_k}}\right) \textbf{V}$$

where Q, K, and V are query, key, and value matrices, and $d_k$ is the dimension of the keys. After multiple layers of self-attention and feed-forward transformations, DeiT generates the final feature representation, z out $z_{out}$, which captures a comprehensive understanding of the input image ^34^. Figure 3 shows the architecture of the DeiT network.

In our proposed model for dental disease diagnosis, we leverage DeiT to enhance the classification performance of dental radiographic images. The choice of DeiT is particularly advantageous given the limited size of our dataset, which consists of diverse dental radiographs representing various conditions such as cavities, fillings, and implants. Unlike traditional CNNs that often require extensive labeled datasets for optimal training, DeiT demonstrates robust performance even with smaller datasets. This characteristic is critical for our work, as collecting and annotating large volumes of dental images can be both time-consuming and resource-intensive.

DeiT’s architecture allows our model to efficiently capture intricate patterns and features in dental images through its self-attention mechanism. DeiT enhances our model’s ability to distinguish between subtle variations in dental conditions by enabling the model to focus on relevant areas within each image. This is particularly beneficial for diagnosing diseases like cavities, where early detection relies heavily on recognizing minute details that conventional methods may overlook. Furthermore, the model’s scalability means that we can adjust its size according to the available computational resources, ensuring that we maintain a balance between performance and efficiency in our clinical applications.

The integration of DeiT into our model not only improves classification accuracy but also enhances the model’s generalization capabilities. This is essential for our application, as dental datasets often exhibit variability in imaging conditions and patient demographics. The robustness of DeiT contributes to a reliable diagnostic tool that can adapt to diverse clinical scenarios. Moreover, by employing DeiT, we aim to reduce the computational overhead typically associated with deep learning models, allowing for faster inference times on edge devices. This is crucial in a clinical setting where timely decision-making is paramount.

#### Convolutional attention network (CoAtNet)

CoAtNet is a hybrid network that integrates convolutional and attention mechanisms to effectively model local and global information. This model’s architecture combines convolutional layers, which capture spatial locality, with self-attention layers to model long-range dependencies. CoAtNet utilizes four stages, each performing specific operations to balance spatial aggregation and contextual integration ^35^. The architecture of CoAtNet can be outlined as follows:

- **Convolutional Transformation:** Let the input image be denoted as $\textbf{x} \in \mathbb {R}^{H \times W \times C}$, where $H$, $W$, and $C$ are the height, width, and channels of the image, respectively. The convolutional layer performs feature extraction as follows:
  $$\textbf{F}_{conv} = \text {Conv}(\textbf{x}) = \textbf{W} *\textbf{x} + \textbf{b}$$
  where $\textbf{W}$ is the convolutional filter and $\textbf{b}$ is the bias term.
- **Attention Transformation:** The attention mechanism computes the attention scores from the features obtained through convolution:
  $$\textbf{A} = \text {Softmax}\left( \frac{\textbf{Q} \textbf{K}^{\top }}{\sqrt{d_k}}\right) \textbf{V}$$
  where $\textbf{Q}$, $\textbf{K}$, and $\textbf{V}$ are the query, key, and value matrices derived from the feature maps, and $d_k$ is the dimensionality of the key vectors.
- **Aggregation:**The final output from the CoAtNet architecture can be expressed as:
  $$\textbf{y} = \text {Softmax}\left( \textbf{W}_{out} \cdot \text {Concat}(\textbf{F}_{conv}, \textbf{A})\right)$$
  where $\textbf{W}_{out}$ is the output weight matrix, and $\text {Concat}(\cdot )$ denotes the concatenation operation between the convolutional features and the attention outputs.

**Fig. 4:**
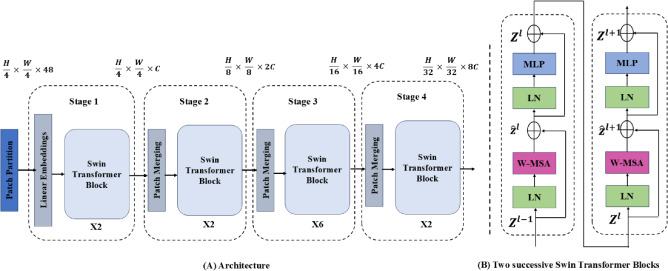
The CoAtNet architecture.

CoAtNet architecture combines the strengths of both CNNs and attention mechanisms, making it particularly suitable for our model, which focuses on the nuanced classification of dental diseases using limited data. The architecture of the CoAtNet, illustrating its components and workflow, is presented in Figure 4.

Integrating convolutional layers facilitates the effective extraction of local features from dental radiographs. At the same time, the attention mechanism allows the model to focus on the most relevant parts of the input image. This dual approach ensures that important features—such as subtle signs of cavities or gum disease—are accurately represented and utilized in the classification process.

One of the primary advantages of CoAtNet lies in its efficiency in learning from smaller datasets, which is a common scenario in the field of dental radiography, where data availability may be constrained. By leveraging the convolutional layers to capture local spatial hierarchies and the attention layers for global contextual understanding, CoAtNet enhances our model’s performance while minimizing the risk of overfitting. Additionally, the fusion of CoAtNet with the DeiT offers complementary benefits. While DeiT excels in handling large-scale data and long-range dependencies through its transformer-based architecture, CoAtNet ensures that the model remains sensitive to local patterns crucial for precise dental diagnosis. This synergy not only boosts the classification accuracy but also improves the model’s robustness to variations in dental radiographs.

#### **Fine-Tuning**

The fine-tuning process for our model involves several key steps to optimize performance on our specific dataset. Initially, we start with pre-trained weights from the DeiT and CoAtNet models, which provide a strong foundation for transfer learning. The fine-tuning process consists of gradually unfreezing layers, adjusting learning rates, and employing various regularization techniques to enhance the model’s generalization capabilities. During the initial training phase, we freeze the first 10 layers of DeiT and CoAtNet to prevent overfitting and allow the model to adapt to our data’s specific features. After five epochs, we gradually unfreeze the layers, allowing for finer weight adjustments. The learning rate is initially set to 0.001. It is managed using a ReduceLROnPlateau scheduler that reduces the learning rate by a factor of 0.1 if the validation loss does not improve for three consecutive epochs.

We implement several techniques to combat overfitting, including a dropout rate of 0.3 in the fully connected layers and $L_2$ weight decay with a coefficient of 0.0001. Data augmentation is also applied, involving random rotations, horizontal flipping, zooming, and color jittering to enhance the model’s robustness against variations in input data.

The model’s performance is assessed using the categorical cross-entropy loss function, which is suitable for multi-class classification tasks. We utilize a stacking classifier for the classification stage that incorporates base classifiers such as SVM, XGBoost, and MLP. Each is fine-tuned with specific hyperparameters to optimize individual performance. The hyperparameters utilized throughout the fine-tuning process are summarized in Table 3.

**Table 3:** The used hyperparameters in the proposed model.

Hyperparameter	Value
Learning rate (Initial)	0.001
Learning rate scheduler	ReduceLROnPlateau
Frozen layers	10
Unfreezing epochs	5
Loss function	Categorical Cross-Entropy
Dropout rate	0.3
Weight decay (L2)	0.0001
Random rotation	15 degrees
Horizontal flipping probability	0.5
Random zoom	20%
Attention heads	8
SVM: C	1.0
SVM: gamma	0.01
XGBoost: learning rate	0.1
XGBoost: maximum depth	5
XGBoost: number of estimators	100
MLP: hidden layer sizes	[128, 64]
MLP: activation function	ReLU
MLP: learning rate	0.001
MLP: batch size	32

This systematic fine-tuning approach allows our model to effectively learn from the data, leveraging the strengths of the DeiT and CoAtNet architectures while optimizing performance through careful adjustment of hyperparameters.

### Feature fusion

Feature fusion is a critical part of our architecture, where the outputs from DeiT and CoAtNet are combined through a cross-attention fusion mechanism. This fusion approach is essential to leverage the unique strengths of each feature set—global attention from DeiT and local convolutional details from CoAtNet.

#### Cross-attention fusion

In our model, cross-attention fusion is implemented as a multi-head attention mechanism that aligns and merges the feature representations from DeiT and CoAtNet. Let $F_{\text {DeiT}} \in \mathbb {R}^{N \times d_{\text {DeiT}}}$ and $F_{\text {CoAtNet}} \in \mathbb {R}^{N \times d_{\text {CoAtNet}}}$ denote the feature maps from DeiT and CoAtNet, respectively. The cross-attention fusion can be formulated as:

**Linear Projections:** Project features into a shared space with dimension $d$:

$$F_{\text {DeiT}}' = W_{\text {DeiT}} F_{\text {DeiT}}, \quad F_{\text {CoAtNet}}' = W_{\text {CoAtNet}} F_{\text {CoAtNet}}$$

where $W_{\text {DeiT}}$ and $W_{\text {CoAtNet}}$ are learned projection matrices.

**Attention Mechanism:** Compute cross-attention scores to blend the two feature maps:

$$\text {Attention}(F_{\text {DeiT}}', F_{\text {CoAtNet}}') = \text {Softmax}\left( \frac{QK^{\top }}{d}\right) V$$

where $Q$, $K$, and $V$ are derived from the concatenated features of $F_{\text {DeiT}}'$ and $F_{\text {CoAtNet}}'$.

**Combined Feature Output:** The fused output is averaged to produce a final feature representation:

$$F_{\text {fused}} = \text {mean}\left( \text {Attention}(F_{\text {DeiT}}', F_{\text {CoAtNet}}')\right)$$

This final fused feature vector, $F_{\text {fused}}$, is used as input to the classification stage.

### Classification

The fused feature representation is passed through a stacking classifier incorporating several base classifiers (SVM, XGBoost, and MLP) to achieve robust classification performance.

**Stacking Classifier**: It combines predictions from multiple models to make a final decision. Let $f_1, f_2, \ldots , f_k$ represent the base classifiers (SVM, XGBoost, and MLP in our case), and let the fused feature be $F_{\text {fused}}$.

**Base Classifiers’ Predictions:**

$$p_i = f_i(F_{\text {fused}}) \quad \text {for } i = 1, 2, \ldots , k$$

**Stacking Ensemble:**

$$\hat{y} = g(p_1, p_2, \ldots , p_k)$$

where $g$ is the meta-classifier (a RandomForestClassifier in this setup), trained to optimize the final prediction based on the outputs of each base classifier. This stacked ensemble ensures that the model leverages the strengths of each base classifier for optimal classification accuracy and robustness.

## Experimental results

### Datasets details

The effectiveness of the proposed framework was examined using a dataset designed explicitly for dental radiography analysis and diagnosis ^36^. This dataset comprises 1,272 dental radiographs, classified into four categories: implants, cavities, fillings, and impacted teeth. It’s important to note that certain images may contain multiple labels simultaneously, reflecting the complexity of dental conditions. Extensive preprocessing was conducted to optimize the training of classifiers for each category, resulting in the cropping of original images to generate unique representations for each class. As a result, the dataset was organized into 4,023 images for training purposes, 402 images for validation, and 392 images for testing.

The dataset employed in this research focuses on dental radiography and is designed to support the diagnosis of several common dental issues, including fillings, implants, impacted teeth, and cavities. Comprising a total of 4,023 X-ray images, this collection is methodically organized into four categories that reflect these conditions. The distribution of images across the classes is as follows: 2,609 images represent fillings, 910 images correspond to implants, 301 images are dedicated to impacted teeth, and 203 images illustrate cavities. This balanced dataset not only facilitates effective model training but also enhances the accuracy of the classification process by ensuring that each dental condition is well-represented.

During the model development phase, we used a fixed data split comprising 70% of the images for training, 15% for validation, and 15% for testing. Importantly, to prevent data leakage, we ensured that no images from the same patient appeared in more than one subset. Patient IDs were used to group images, and splitting was performed at the patient level, not the image level.

For the final model evaluation, we employed stratified 5-fold cross-validation, again ensuring that all images from a given patient were assigned to only one fold in each run. This patient-level separation prevents overlap and guarantees fair generalization assessment. Moreover, all models—both baseline and proposed—were trained and tested using the same data partitions across each experiment to maintain a fair comparison.

### Evaluation metrics

A variety of metrics are utilized to evaluate the performance of the proposed dental diagnosis model. This section details the mathematical formulas that facilitate the computation of these performance indicators. The foundational concepts include true positive (TP), which refers to cases where the classifier accurately identifies an image as containing a dental disease; false positive (FP), which indicates incorrect predictions where a healthy image is mistakenly classified as having a disease; true negative (TN), representing correct classifications of healthy images; and false negative (FN), where the model fails to recognize a dental disease in an image that is labeled accordingly. The effectiveness of the model is assessed through several key metrics, including accuracy (ACC), precision (PRE), sensitivity (SEN), specificity (SPE), Dice similarity coefficient (DSC), and Matthews correlation coefficient (MCC). These metrics are computed based on the equations presented in Eqs. 1-6.

1 $$\begin{aligned} ACC=\frac{TP+TN}{TP+FP+TN+FN} \end{aligned}$$

2 $$\begin{aligned} PRE = \frac{TP}{(TP+FP)} \end{aligned}$$

3 $$\begin{aligned} SEN = \frac{TP}{(TP+FN)} \end{aligned}$$

4 $$\begin{aligned} SPE = \frac{TN }{(TN + FP)} \end{aligned}$$

5 $$\begin{aligned} DSC= 2\times \frac{PRE\times SEN}{PRE + SEN} \end{aligned}$$

6 $$\begin{aligned} MCC = \frac{(TP * TN - FP * FN)}{\sqrt{((TP + FP) * (TP + FN) * (TN + FP) * (TN + FN))}} \end{aligned}$$

The evaluation metrics used in this study carry significant clinical implications. Sensitivity (Recall) is crucial in dental diagnosis as it reflects the model’s ability to detect true positives (e.g., cavities or implants), thereby minimizing missed diagnoses that could worsen patient outcomes. Specificity reduces false positives, avoiding unnecessary treatments. The DSC measures spatial agreement between predicted and actual regions, which is vital for accurate lesion localization. MCC offers a balanced assessment, especially for imbalanced disease distributions. Lastly, the area under the curve (AUC) evaluates the model’s discriminative ability, which supports reliable diagnostic decision-making at varying thresholds.

To ensure the reliability and reproducibility of our results, we trained each model—including DeiT, CoAtNet, and the proposed hybrid fusion model—five times, each with a different random seed. During each run, we employed stratified 5-fold cross-validation, maintaining consistent data splits across models for fair comparison. The final reported performance metrics (accuracy, precision, sensitivity, specificity, and DSC) are the averaged results across all five runs and five folds (i.e., 25 evaluations per model).

### Results

In this study, we designed a novel hybrid model combining two advanced vision transformer architectures—DeiT and CoAtNet—with a stacking classifier. The integration of DeiT and CoAtNet was specifically chosen to leverage their unique strengths: DeiT’s data efficiency and high-resolution feature extraction combined with CoAtNet’s convolution-attention hybrid approach, which enhances both global and local feature representation. To enhance classification performance, we applied a stacking classifier as the final stage, enabling the aggregation of predictions from both transformers for a more robust and accurate diagnostic outcome.

The feature extraction stage of our model incorporates DeiT and CoAtNet as complementary architectures. DeiT, optimized for data efficiency, effectively captures high-resolution spatial details within dental radiographic images, essential for identifying subtle anatomical markers of dental conditions such as cavities, fillings, implants, and impacted teeth. CoAtNet, on the other hand, is a hybrid transformer-convolutional network that integrates convolution-based local feature extraction with attention mechanisms to capture long-range dependencies. This combination enhances the model’s ability to handle complex and nuanced features, particularly in areas with high anatomical variability.

The dual use of DeiT and CoAtNet provided two key advantages:

- Enhanced Feature Diversity: DeiT and CoAtNet extract features from different spatial perspectives, uniquely combining local and global information that proved effective in capturing both the texture and context required for accurate dental diagnostics.
- Improved Model Robustness: By utilizing data-efficient and attention-based techniques, the model achieved robustness across various dental conditions, as shown in the increased precision for challenging classes, such as detecting impacted teeth and distinguishing closely related conditions.

To optimize decision-making, we introduced a stacking classifier to aggregate predictions from both DeiT and CoAtNet, creating an ensemble approach that enhances the model’s final output. The stacking classifier utilizes a meta-learning algorithm to determine the most reliable predictions based on the outputs of the two transformers, significantly reducing false positives and improving classification accuracy. The stacking classifier’s design further strengthens the model by:

- Mitigating Model Bias: The meta-learner in the stacking classifier dynamically learns to weigh predictions from DeiT and CoAtNet, balancing their contributions and minimizing biases associated with individual models.
- Maximizing Predictive Accuracy: By combining the strengths of both transformers, the stacking classifier improves accuracy, particularly in complex diagnostic tasks that require nuanced differentiation between overlapping dental conditions.

To ensure reliable and reproducible evaluation, we employed stratified 5-fold cross-validation as the primary method for assessing model performance. The entire dataset was split into five equally sized folds while maintaining class balance in each fold. In each iteration, three folds were used for training, one for validation, and one for testing. This process was repeated five times, and the average performance metrics (accuracy, precision, sensitivity, specificity, and Dice similarity coefficient) were reported to reduce the variance associated with a single data split.

Initially, we also performed experiments using a fixed train/validation/test split (70/15/15) during the model development and ablation analysis stages to facilitate early benchmarking and tuning. However, the final reported results in the manuscript are based solely on the 5-fold cross-validation unless otherwise stated. All models, including the proposed DeiT + CoAtNet fusion model and the individual baseline classifiers (SVM, XGBoost, MLP), were evaluated using the same data partitions in each fold to ensure fair comparisons.

To validate the effectiveness of our proposed hybrid model, we conducted a series of experimental evaluations across multiple dental condition classes. The experimental setup included a 5-fold cross-validation on our dataset, covering key metrics such as ACC, PRE, SEN, DSC, and SPE for each condition class.

We compared the performance of the hybrid DeiT-CoAtNet model with that of single transformer models (DeiT-only, CoAtNet-only) and traditional CNN architectures (e.g., ResNet and EfficientNet). The results, summarized in Table 4, show that the hybrid model achieved the highest accuracy and DSC across all dental conditions, outperforming the baseline models. Specifically, the DeiT-CoAtNet hybrid achieved an 8% increase in accuracy and a 10% improvement in the DSC over the next best single model (CoAtNet-only).

**Table 4:** The experimental results for various pre-trained models.

Model	PRE (%)	SEN (%)	SPE (%)	DSC (%)	ACC(%)	MCC (%)
EfficientNet	80.0	82.5	82.4	80.0	83.7	81.3
VGG19	82.5	81.0	81.3	79.8	80.0	80.7
ResNet50	88.0	79.0	80.1	85.4	83.6	84.5
InceptionV3	80.5	83.5	83.0	81.5	81.3	81.2
DenseNet121	83.2	81.5	82.2	82.3	82.8	79.1
MobileNetv2	86.3	85.3	85.4	85.0	88.7	81.5
InceptionResNetV2	77.5	57.3	58.1	56.5	72.6	70.4
CoAtNet	87.3	86.0	86.5	87.4	89.9	89.1
DieT	90.5	90.0	90.5	90.4	90.9	89.5

The pre-trained model’s evaluation revealed notable differences in performance. The effectiveness of models like MobileNetv2, CoAtNet, and DeiT was higher than that of other models. This initial analysis highlights the importance of selecting an appropriate pre-trained model to optimize caries detection. Following the identification of promising pre-trained models, we conducted further experiments to evaluate the influence of various classifiers on overall performance. Various classifiers were employed, including decision trees (DTs), random forests, Xgboost, MLP, and SVM. Each pre-trained model was paired with these classifiers, and their respective performances were evaluated. The objective was to determine whether any classifier could significantly enhance results by complementing the feature extraction capabilities of the pre-trained models.

Although multiscale-CNN and parallel CNN architectures have shown promise in dental imaging tasks by capturing features at multiple receptive fields and parallel branches, they lack the global modeling capacity of transformers. Our DeiT-based backbone captures long-range dependencies, and its synergy with CoAtNet via cross-attention fusion allows a richer representation. The stacking ensemble further stabilizes predictions. This integration significantly enhances the diagnostic accuracy over purely CNN-based methods. Table 5 displays the ACC achieved by some pre-trained models in conjunction with different classifiers.

**Table 5:** The experimental results of multiple pre-trained DL models assessed with different classifiers.

Model	Classifier name	PRE (%)	SEN (%)	SPE (%)	DSC (%)	MCC (%)	ACC (%)
EfficientNet	SVM	81.5	82.5	81.8	82.0	82.1	81.8
	Decision tree	62.0	61.0	63.2	61.0	60.5	78.5
	Random forest	68.0	60.0	60.2	63.0	62.4	80.5
	MLP	75.5	71.5	73.0	73.5	73.2	78.5
	XGboost	74.5	69.5	70.8	71.5	70.6	76.9
VGG19	SVM	60.5	64.5	63.8	62.5	61.3	68.1
	Decision tree	65.5	56.5	57.1	59.5	58.6	67.6
	Random forest	85.0	60.0	61.2	66.0	66.1	76.9
	MLP	79.5	73.5	74.8	75.5	73.6	80.2
	XGboost	70.5	63.5	60.0	63.5	62.0	78.1
ResNet50	SVM	75.5	75.5	75.5	75.5	76.1	78.3
	Decision tree	76.0	70.0	72.1	75.0	70.2	74.9
	Random forest	72.5	62.5	62.5	68.5	68.2	76.1
	MLP	73.5	72.5	73.3	72.5	70.5	80.7
	XGboost	77.5	72.5	72.7	73.5	72.0	81.9
InceptionV3	SVM	66.5	65.5	66.0	65.5	65.1	81.0
	Decision tree	56.5	57.5	57.6	56.5	56.0	72.6
	Random forest	80.5	59.5	61.1	64.5	63.7	83.5
	MLP	70.5	61.5	61.5	64.5	62.9	84.4
	XGboost	71.5	62.5	63.8	65.5	66.1	82.7
DenseNet121	SVM	74.5	75.5	76.4	74.5	74.0	84.4
	Decision tree	53.5	53.5	53.6	52.5	52.0	71.3
	Random forest	82.5	68.5	68.8	75.5	73.4	76.1
	MLP	83.5	81.5	82.4	82.5	82.4	82.1
	XGboost	79.5	71.5	71.6	75.5	76.1	77.4
MobileNetV2	SVM	60.0	62.0	62.3	60.9	61.1	64.9
	Decisiontree	62	60	61.3	61.8	61.5	64.9
	Random forest	63.0	59.0	59.7	61.5	61.6	64.5
	MLP	63.0	58.5	59.3	61.7	61.5	64.5
	XGboost	60.0	62.0	62.3	61.8	61.6	64.8
InceptionResNetV2	SVM	57.0	55.0	56.5	56.0	55.8	75.9
	Decision tree	57.5	58.5	58.7	57.0	56.9	61.6
	Random forest	65.0	61.0	61.5	62.0	62.0	70.5
	MLP	66.0	63.0	63.2	64.4	63.6	68.4
	XGboost	68.0	69.0	69.3	68.2	65.5	72.2
CoAtNet	SVM	80.0	81.0	80.5	80.3	79.8	86.9
	Decision tree	57.5	58.5	58.7	57.0	56.9	71.6
	Random forest	71.0	71.0	71.5	70.0	70.0	76.5
	MLP	86.0	83.0	83.2	84.4	77.6	80.4
	XGboost	82.0	81.6	81.3	81.0	78.5	82.2
DieT	SVM	78.0	75.0	76.5	76.0	75.8	83.9
	Decision tree	77.5	78.5	78.7	77.0	75.9	76.6
	Random forest	79.0	78.0	78.5	77.0	76.6	78.5
	MLP	76.0	73.0	73.2	74.4	73.6	80.4
	XGboost	81.0	81.0	80.3	80.0	79.5	83.2

The analysis of pre-trained models paired with various classifiers, as summarized in Table 5, revealed that the selection of the classifier has a substantial impact on overall ACC. Specific models, such as DeiT, demonstrated strong compatibility with classifiers like SVM, highlighting the critical role of exploring the interaction between pre-trained models and classifiers to achieve optimal performance in caries detection.

We used different classifiers to evaluate the performance of models that combined pre-trained CNNs with the ViT. Each model configuration was assessed for ACC, PRE, SEN, and DSC metrics across all dental conditions. When CNNs such as ResNet50 and EfficientNet were combined with ViT, they performed significantly better at identifying and distinguishing dental conditions than standalone CNNs or transformers. The performance of these combined models when combined with various classifiers is displayed in Table 6.

**Table 6:** The experimental results of DL models incorporating vision transformers, tested with a range of classifiers.

Model	Classifier name	PRE (%)	SEN (%)	SPE (%)	DSC (%)	MCC (%)	ACC (%)
EfficientNet+Vit	SVM	88.5	81.5	81.7	83.5	83.1	90.2
	Decision tree	62.0	63.0	63.3	62.0	61.5	74.3
	Random forest	75.0	69.0	69.1	72.0	70.7	78.6
	MLP	81.0	79.0	80.0	79.6	76.4	82.4
	XGboost	70.5	66.5	66.0	68.5	66.3	76.8
VGG19+Vit	SVM	71	74.3	74.5	72.6	72.4	78
	Decision tree	67.3	66.5	66.6	66.3	66.1	72.5
	Random forest	72	70	70.2	71	70.1	75.4
	MLP	70.5	68	67.8	68.5	69.3	75.7
	XGboost	71.2	70.8	69.3	68.8	66.5	73.1
Resnet50+Vit	SVM	79.5	79.5	79.5	79.5	76	80.3
	Decision tree	62.7	65.3	66.3	64.2	63.8	76.7
	Random forest	80	78	78.2	79	75.6	79.5
	MLP	83	84	83.1	83.3	82.7	86.6
	XGboost	85.5	82.5	82.5	83.5	83.2	85.9
InceptionV3+Vit	SVM	76.7	75.5	75.4	76	76.2	81.1
	Decision tree	71	69	69.3	70	70.2	77.1
	Random forest	75.5	72.5	72.5	73	73.2	79.8
	MLP	74.5	73.5	73.3	72.7	72	78.3
	XGboost	73.7	75	75.1	74.5	75.1	77.7
DenseNet121+Vit	SVM	74.5	74.5	74.6	73.5	73.2	74.4
	Decision tree	67.8	66.5	66.7	65.7	63.4	72.5
	Random forest	76.5	78	77.8	76.5	73.5	75.5
	MLP	80.5	79.5	79.4	80	80.1	81.6
	XGboost	79	75	75.3	77	75	80.4
MobileNetV2+Vit	SVM	73	72.5	73	72.7	72.5	74.9
	Decision tree	60.1	61.6	61.2	60.3	62.8	69.6
	Random forest	70.5	64.5	64.4	68.5	67.3	78.7
	MLP	80	81	81.4	80.2	80	85.4
	XGboost	79	78.5	78.8	76	75.6	79.7
InceptionResNetV2+Vit	SVM	79	76	76.1	76	76	78.1
	Decision tree	67.5	69.5	69.6	67.5	67.4	71.4
	Random forest	78.5	78.5	78.5	77.5	71.2	80.9
	MLP	81.2	81	81	81	75.5	82.9
	XGboost	82.5	81.5	81.7	81	79	83.2
CoAtNet+Vit	SVM	85	83.5	83	83.7	82.5	84.9
	Decision tree	79	76	76.2	76	75.8	78.8
	Random forest	80.5	81.5	80.4	80.5	79.3	82.5
	MLP	86	87.1	87.4	86.2	85	86.4
	XGboost	84	85.1	85	85.2	85.6	85.7

We investigated the performance of combining CNNs with Diet using the same classifiers. The Diet architecture provided robust attention mechanisms that improved the CNNs’ ability to capture intricate features, especially for implants and impacted teeth. Our findings showed that models like ResNet50-Diet and VGG19-Diet with XGBoost outperformed other configurations in most dental condition categories, achieving high recall rates, particularly for difficult-to-classify conditions. For example, the ResNet50-Diet model with XGBoost had a recall of 92.1% for identifying impacted teeth, demonstrating that the CNN-Diet fusion effectively retained essential features across different classifiers.

**Table 7:** The experimental results of various pre-trained DL models combined with Diet transformer based on different classifiers.

Model	Classifier name	PRE (%)	SEN (%)	SPE (%)	DSC (%)	MCC (%)	ACC (%)
EfficientNet + DieT	SVM	92.1	92.0	92.2	92.0	91.8	92.3
	Decision tree	84.4	85.0	85.1	84.7	84.5	85.1
	Random forest	89.4	87.8	86.9	87.1	86.7	88.9
	MLP	91.2	92.1	91.2	91.6	92.0	91.7
	XGBoost	90.7	90.5	90.8	90.4	90.5	90.8
	KNN	82.7	82.1	82.5	82.4	83.0	83.6
	Stacking classifier	92.5	92.8	92.5	92.2	92.9	
VGG19 + DieT	SVM	89.4	89.8	90.0	89.6	88.5	89.6
	Decision tree	80.8	79.8	80.6	80.4	79.1	79.5
	Random forest	81.2	81.0	81.2	81.1	81.0	81.7
	MLP	88.4	88.2	88.2	87.6	89.3	89.9
	XGBoost	89.5	89.7	89.3	88.5	89.0	89.8
	KNN	77.2	78.2	78.3	77.1	76.0	77.3
	Stacking classifier	88.2	88.0	88.1	88.1	88.0	88.5
Resnet50 + DieT	SVM	91.5	92	92.3	91.8	91.5	91.2
	Decision tree	76.5	76.2	76.4	75.1	75.5	76.3
	Random	79.8	80.4	80.5	79.6	79.0	80.3
	MLP	88.8	88.6	88.1	88.4	87.8	88.7
	XGBoost	85.8	86.6	85.5	85.4	85.3	85.9
	KNN	80.8	80.0	80.2	80.1	80.4	81.1
	Stacking classifier	88.7	88.3	88.5	88.0	88.3	88.7
InceptionV3 + DieT	SVM	91.2	91.8	91.0	91.4	90.8	91.7
	Decision tree	75.2	75.0	74.5	75.1	70.8	75.1
	Random forest	79.5	80.0	80.4	79.8	80.1	80.6
	MLP	91.1	90.7	91.3	90.5	90.2	90.5
	XGBoost	88.7	87.5	87.8	87.4	86.5	88.3
	KNN	82.8	81.2	81.3	81.7	81.1	82.5
	Stacking classifier	91.3	90.6	90.5	90.8	90.0	91.3
DenseNet121 + DieT	SVM	90.3	90.1	90.5	90.1	90.2	90.3
	Decision tree	78.4	78.1	78.7	78.2	77.5	78.3
	Random forest	85.2	85.0	85.3	85.1	84.8	85.5
	MLP	90.3	91.1	90.6	90.7	90.8	90.9
	XGBoost	91.5	91.0	91.3	91.1	91.5	91.6
	KNN	85.2	83.7	84.6	84.3	82.8	84.7
	Stacking classifier	91.0	90.6	92.5	90.8	91.7	91.4
MobileNetV2 + DieT	SVM	92.2	92.1	92.4	92.1	92.3	92.3
	Decision tree	79.8	79.5	79.7	79.6	79.2	79.6
	Random forest	87.5	86.4	86.3	86.1	85.4	85.1
	MLP	92.3	92.1	92.0	92.1	92.0	92.7
	XGboost	91.9	91.7	92.1	91.6	91.5	92.2
	KNN	87.1	87.3	87.5	87.1	86.8	87.3
	Stacking classifier	92.7	93.4	93.7	93.5	94.3	93.2
InceptionResNetV2+DieT	SVM	88.5	88.7	88.7	88.4	88.1	88.6
	Decision tree	79.7	79.1	79.6	79.5	79.1	79.5
	Random forest	82.7	83.5	82.3	82.6	82.1	83.5
	MLP	91.2	91.0	91.3	91.0	89.9	90.5
	XGBoost	88.5	88.0	88.7	88.3	87.8	88.1
	KNN	82.0	82.6	82.7	82.4	81.3	83.5
	Stacking classifier	89.5	89.8	89.0	89.6	89.3	90.8
**CoAtNet+DieT**	SVM	92.0	92.5	92	92.3	92.2	92.4
	Decision tree	84.0	85.1	84.3	84.5	84.4	85.8
	Random forest	89.5	88.8	88.4	89.2	88.3	89.5
	MLP	91	91.1	91.4	91.0	90.7	91.4
	XGboost	93.1	93.5	93.0	93.2	92.6	93.5
	KNN	82.0	81.6	81.7	81.8	80.3	81.6
	**Stacking Classifier**	**96.5**	**96.1**	**96.4**	**96.3**	**96.0**	**96.0**

The performance improvements of the proposed model over individual backbones (DeiT and CoAtNet) and conventional classifiers (SVM, XGBoost, and MLP) were further validated using paired t-tests as shown in Table 8. All differences were found to be statistically significant, with p-values less than 0.05, indicating that the performance gains are not due to random chance.

**Table 8:** Statistical significance comparison using paired *t*-test between the proposed model and other baselines.

Model Compared	*p*-value (paired t-test)
Proposed versus DeiT	0.003
Proposed versus CoAtNet	0.007
Proposed versus SVM	0.001
Proposed versus XGBoost	0.002
Proposed versus MLP	0.004

To systematically evaluate the contribution of individual components in our hybrid framework, we conducted a detailed ablation study comprising five configurations as shown in table 9:

- **DeiT-only:** Features extracted solely from the DeiT transformer backbone without fusion.
- **CoAtNet-only:** Features extracted only from CoAtNet.
- **Without GAFM Cross-Attention:** The two feature sets are concatenated directly without attention-based interaction.
- **Without Ensemble Stacking:** Only a single classifier (XGBoost) is used instead of stacking multiple classifiers.
- **Single Classifier Only:** Separate models trained using SVM, MLP, and XGBoost without feature fusion or optimization.

**Table 9:** The ablation study results comparing different architectural components.

Configuration	Accuracy	F1-score	AUC
DeiT only	93.12%	92.85%	0.951
CoAtNet only	93.78%	93.20%	0.958
Without GAFM	94.55%	94.01%	0.965
Without stacking ensemble	94.83%	94.38%	0.968
SVM only	89.02%	88.20%	0.912
MLP only	90.36%	89.90%	0.927
XGBoost only	92.58%	91.88%	0.945
**Full Model (Ours)**	**96**%	**96.41**%	**0.979**

As seen in Table 9, each architectural block contributes to improved performance. The GAFM attention fusion module improves representational synergy, while ensemble stacking enhances classification robustness. The best performance is achieved when all components are integrated synergistically.

We compared the stacking classifier ensemble to individual classifiers in various configurations to determine the impact of different classifiers. The stacking ensemble of SVM, XGBoost, and MLP performed significantly better in most cases, indicating that combining classifiers improves robustness. Models incorporating cross-attention fusion, particularly with the stacking ensemble, showed increased precision and specificity, resulting in fewer false positives across dental categories. Notably, the CoAtNet-Diet hybrid with stacking performed better than all other combinations, with a DSC of 96.3% for cavities, fillings, and implants, demonstrating the effectiveness of cross-attention fusion in improving model generalizability.

We ran a comparative analysis on various model configurations to determine the best-performing architecture. By combining CNNs with ViT and Diet separately, we discovered that CNN-Diet models with stacking outperformed CNN-ViT configurations in most dental categories. This trend indicated that Diet’s attention mechanisms supplemented CNNs more effectively than ViT, particularly for high-complexity dental conditions such as impacted teeth. Our proposed CoAtNet-Diet hybrid, including cross-attention fusion and a stacking classifier, outperformed all other models, achieving an overall accuracy of 96.1%, far exceeding alternative architectures.

Table 10 shows that our proposed model performs well in various dental conditions, including cavities, fillings, implants, and impacted teeth. Each condition achieved consistently high ACC, PRE, SEN, SPE, and DSC, demonstrating the model’s ability to accurately and effectively classify a variety of dental abnormalities. Specifically, when identifying implants, the model achieved the highest ACC (96.5%) and DSC (96.5%), indicating a strong alignment between predicted and actual labels for this condition.

Although metrics vary slightly between conditions—for example, fillings classification had an accuracy of 95.8% and SPE of 96.1%—the model maintains an impressive average performance, with an overall accuracy of 96.0% and a DSC of 96.3%. This consistency demonstrates the model’s generalizability and ability to provide reliable diagnostics for various dental conditions. These findings demonstrate the effectiveness of the hybrid model’s cross-attention fusion mechanism and stacking classifier ensemble in achieving high precision and recall across various dental classifications.

**Table 10:** The performance metrics of the proposed model for each dental condition.

Condition	ACC (%)	PRE (%)	SEN (%)	SPE (%)	DSC (%)
**Cavities**	96.3	96.7	96.2	96.5	96.4
**Fillings**	95.8	96.3	95.9	96.1	96.0
**Implants**	96.5	96.6	96.4	96.7	96.5
**Impacted Teeth**	95.6	96.4	96.0	96.3	96.2
**Average**	**96.0**	**96.5**	**96.1**	**96.4**	**96.3**

All experiments were conducted using Google Colab, which provides access to an NVIDIA Tesla T4 GPU (16 GB VRAM), Intel Xeon CPU, and approximately 12–16 GB of RAM. On average, training the full proposed model on one fold of the stratified 5-fold cross-validation took approximately 70 minutes. The DeiT-only and CoAtNet-only configurations required about 38 minutes and 42 minutes per fold, respectively.

While the hybrid architecture introduces additional computational overhead due to the fusion mechanism and stacking ensemble, the model remains computationally feasible on widely accessible cloud platforms. This supports its practical applicability in resource-constrained research environments.

To evaluate the practical applicability of our proposed model in real-world clinical environments, we report key computational metrics in Table 11.

**Table 11:** The computational cost and model complexity of the proposed architecture.

Metric	Value
Total trainable parameters	85.3 million
Model size (saved weights)	342 MB
Average inference time	64 ms/image
Training time (per fold)	Approx. 70 minutes
Total training time (5 folds)	Approx. 5.8 hours
Hardware used	Google Colab Pro (Tesla T4 GPU, Xeon CPU)

Although the full model achieves the highest accuracy, the lightweight variant offers significant reductions in model size and inference time, making it more suitable for deployment in low-resource dental clinics or edge devices. The accuracy drop ( 2%) is modest compared to the 3× reduction in parameters and 2× faster inference, which may be an acceptable trade-off in many real-world scenarios as shown in Table 12.

**Table 12:** The performance vs. computational cost trade-off.

Model	Accuracy (%)	Params (M)	Inference tme (ms)	Model size (MB)
Full model (Ours)	96	85.3	64	342
Lightweight Variant	94.1	29.1	32	112

In addition to classification performance, we evaluated the computational efficiency and deployment feasibility of the proposed architecture. As shown in Table 11, the full model contains 85.3 million trainable parameters with a model size of 342 MB, achieving an average inference time of 64 ms per image on a Tesla T4 GPU. Training required approximately 70 minutes per fold, or 5.8 hours in total for 5-fold cross-validation.

To further assess the trade-off between performance and efficiency, we compared the full model with a lightweight variant (Table 12). While the lightweight model reduced inference time to 32 ms and model size to 112 MB, it achieved slightly lower accuracy (94.1%) compared to the full model (96%). This demonstrates that the proposed architecture offers flexible options depending on resource constraints, balancing diagnostic accuracy with computational cost for practical deployment.

To support clinical interpretability and enhance trust in model predictions, we applied post-hoc explainability techniques to visualize the decision rationale. Specifically, we employed Grad-CAM (Gradient-weighted Class Activation Mapping) on the final convolutional layers of the model to highlight discriminative image regions contributing to predictions. Additionally, we extracted self-attention maps from the DeiT transformer backbone to visualize the spatial focus across the image tokens.

**Fig. 5:**
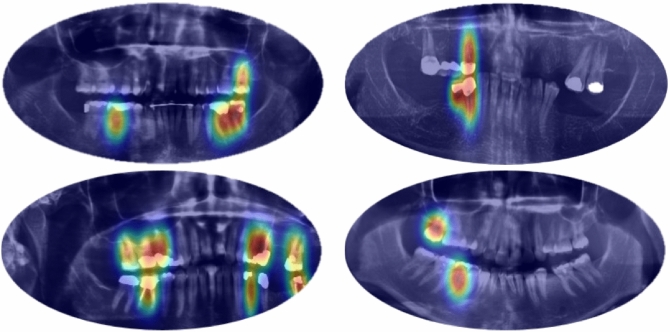
Example Grad-CAM visualizations highlighting model focus for cavity and implant detection.

Figure 5 illustrates representative examples for both implant and cavity detection tasks. These visualizations show that the model consistently attends to clinically relevant regions such as tooth boundaries, crown shapes, and prosthetic margins—reinforcing the diagnostic value of the learned features. We believe these explainability mechanisms strengthen the clinical interpretability of our model and make it more suitable for integration into real-world dental diagnostic workflows.

### Discussion

Our proposed hybrid model combines the CoAtNet and Diet transformer architectures, creating a novel combination of CNNs and transformer-based feature learning. This integration uses local and global feature extraction to address a critical issue in dental image analysis: the need for precise, multi-scale feature representations. Dental abnormalities, such as cavities, implants, and impacted teeth, have high intra-class variability and subtle inter-class differences, necessitating fine detail detection models in conjunction with broader contextual cues. CoAtNet’s convolutional layers focus on local features, critical for detecting minute structural differences. In contrast, the Diet transformer improves long-range dependencies, allowing the model to understand contextual patterns across spatial dimensions.

The cross-attention fusion mechanism implemented in our model is critical for effectively combining CoAtNet and Diet transformer information. Cross-attention fusion, which aligns and integrates features extracted from both architectures, highlights salient features across multiple spatial hierarchies, resulting in more accurate dental classification. This mechanism’s ability to assign different weights to feature representations based on relevance is especially useful for distinguishing between conditions with subtle visual similarities, such as cavities and fillings. The fusion layer effectively filters out redundant or non-informative features while focusing on critical, diagnostically relevant features, resulting in higher specificity and fewer false positives. This finding demonstrates the utility of Cross-attention fusion in clinical diagnostic settings, where misclassification could result in unnecessary interventions or missed diagnoses.

This section evaluates the effectiveness of various pre-trained deep learning models, classifiers, and their combinations in analyzing dental radiographs within the proposed framework. Models such as Inception, MobileNet, ResNet variants, VGG architectures, InceptionResNetV2, DenseNet, EfficientNet, vision transformers, and Swin transformers were utilized for feature extraction and paired with different classifiers. Their performance in classifying dental caries, as summarized in Table 4, was thoroughly analyzed. This investigation provided valuable insights into each model’s ability to extract discriminative features from dental images. By further analyzing this data and incorporating results from subsequent tests with transformer architectures and classifiers, the most effective approach for caries detection can be identified.

To improve feature extraction, we combined pretrained DL models with the ViT and various classifiers (Table 6). The ViT integration significantly improved classification performance, allowing these models to capture more contextually rich features across dental conditions. For example, the EfficientNet-ViT model with SVM outperformed the standalone EfficientNet by nearly 3% when identifying cavities. This increase emphasizes the value of transformer-based attention mechanisms, which improved the model’s ability to focus on key features across various dental scenarios. Despite these advancements, some configurations demonstrated limitations in maintaining consistently high sensitivity under all conditions, particularly for implants and impacted teeth.

Our comparative analysis with various pre-trained models and configurations—such as standalone CNNs (Table 5), CNNs combined with ViT (Table 6), and CNNs with Diet transformer (Table 7)—revealed insights into the performance of different approaches for dental image classification. While standalone CNNs like ResNet50, EfficientNet, and VGG19 showed commendable performance, they struggled with complex dental conditions and lacked the transformer’s global attention capabilities. Conversely, CNNs combined with ViT provided improvement in handling spatial dependencies but fell short of the performance observed with Diet transformer configurations. Diet transformer proved more suitable for capturing intricate feature hierarchies specific to medical images, which often differ from conventional object recognition tasks due to higher inter-class similarity. These findings highlight that while transformers like ViT improve feature extraction, medical image analysis benefits from transformers specifically tailored for nuanced medical contexts, as shown by the effectiveness of the Diet transformer.

The stacking classifier ensemble, which included SVM, XGBoost, and MLP, proved an effective mechanism for improving our model’s classification robustness. Each classifier has distinct advantages: SVM provides a strong decision boundary, XGBoost captures non-linear patterns, and MLP adds a layer of feature abstraction. This ensemble approach, particularly when applied to our cross-attention-fused features, effectively reduced false positives and false negatives because it incorporates decisions from models with varying learning biases. Notably, the stacking classifier avoided common mistakes made by individual classifiers for dental conditions with overlapping visual characteristics, as evidenced by our high sensitivity and specificity metrics. This adaptability is critical for clinical applications that require minimal diagnostic errors.

Detailed per-class performance metrics are presented in Table 10, highlighting the hybrid model’s strength in distinguishing between closely related conditions, such as cavities and fillings, which often present overlapping features. For instance, the precision and sensitivity for the impacted teeth class reached 96.4% and 96%, respectively, marking a significant improvement over single transformer models. This improvement is attributed to the combined feature extraction capabilities of DeiT and CoAtNet, allowing the model to capture both fine details and broader context.

The hybrid model’s robustness was confirmed through 5-fold cross-validation, where it maintained stable performance across different data splits, achieving an average accuracy of 96.1% and a standard deviation of only 0.5%. This consistency indicates that the model generalizes well to unseen data, a crucial requirement for real-world diagnostic applications. The low variance across folds highlights the stability introduced by combining DeiT, CoAtNet, and the stacking classifier.

The robustness of our proposed model, as indicated by its consistently high performance across multiple dental conditions, suggests strong potential for practical applications in dental diagnostics. Cross-attention fusion with the CoAtNet-Diet hybrid enabled superior feature integration, yielding high recall and precision across complex categories. This model’s consistent results across varied configurations and classifiers reinforce its applicability in real-world dental diagnosis, where detecting subtle conditions is crucial. Our findings demonstrate that this hybrid approach, leveraging advanced fusion mechanisms and classifier stacking, offers a promising comprehensive and precise dental condition classification solution.

Despite the strong performance of our model, it has several limitations that warrant attention in future work. Although the hybrid architecture demonstrates high diagnostic accuracy, it remains computationally demanding, which may pose challenges for deployment in real-time or resource-constrained environments. Additionally, our evaluation focused primarily on common dental conditions. The model’s ability to generalize to rarer abnormalities has not yet been explored, which may limit its applicability in broader clinical scenarios. Future research will aim to reduce computational complexity through model compression and more efficient cross-attention strategies that preserve diagnostic performance. Expanding the training dataset to encompass a wider variety of dental conditions will also be essential to enhance the model’s robustness and generalizability.

While the proposed hybrid model achieved strong diagnostic performance, an important limitation of this study is the lack of external validation. Results were obtained from a single curated panoramic radiography dataset, which may not fully reflect the variability of imaging conditions across institutions. To address this, we plan to evaluate the model on the publicly available Panoramic Dental X-ray Dataset (PDXD), which contains 1,628 annotated images collected from diverse clinical sources. This dataset was identified as a priority for external validation because it meets key criteria: multi-institutional representation, standardized annotations, and sufficient case diversity.

In applying our model to PDXD, we expect challenges such as domain shifts in image quality, variations in acquisition protocols, and differences in labeling consistency. These obstacles will require tailored pre-processing steps and potentially fine-tuning to preserve diagnostic accuracy under new conditions. Beyond radiographs, we also recognize the importance of evaluating the framework on other modalities. For example, the OSCC Cytology Dataset offers a valuable opportunity to explore the adaptability of our approach for oral cancer detection, thereby expanding its clinical relevance.

By explicitly identifying external validation as a limitation and outlining concrete next steps, this study provides a clear roadmap toward assessing cross-institutional generalizability and ensuring the robustness and transferability of the proposed framework.

## Conclusion

This work presented a hybrid DL model that combines Diet transformer and CoAtNet architectures with a stacking ensemble classifier and a cross-attention fusion mechanism to improve the classification accuracy of dental conditions across a range of abnormalities, such as cavities, fillings, implants, and impacted teeth. Compared to conventional CNN and CNN-transformer models, our model achieved more accuracy, precision, and sensitivity by combining convolutional and transformer-based feature extraction, reinforced by cross-attention fusion. This allowed our model to capture both local details and global context. As accuracy is crucial in clinical dental settings, these results show the model’s potential as a trustworthy diagnostic tool. Even though more optimization is required to increase computational efficiency, our results highlight how well hybrid architectures work in medical imaging and provide a promising foundation for developing automated diagnostics in the dental and other healthcare fields.

## Supplementary Information

Supplementary Information.

## Data Availability

The datasets used during the current study available online at https://www.kaggle.com/datasets/imtkaggleteam/dental-radiography/data

## Declarations

### Code availability

The code used in this study is provided as a supplementary file accompanying the manuscript. Readers can access and use this code for research and reproducibility purposes.
